# Altered Metabolism of Growth Hormone Receptor Mutant Mice: A Combined NMR Metabonomics and Microarray Study

**DOI:** 10.1371/journal.pone.0002764

**Published:** 2008-07-23

**Authors:** Horst Joachim Schirra, Cameron G. Anderson, William J. Wilson, Linda Kerr, David J. Craik, Michael J. Waters, Agnieszka M. Lichanska

**Affiliations:** 1 Institute for Molecular Bioscience, The University of Queensland, Brisbane, Queensland, Australia; 2 CSIRO Mathematical and Information Sciences, Statistical Bioinformatics - Health, New South Wales, Australia; Karolinska Institutet, Sweden

## Abstract

**Background:**

Growth hormone is an important regulator of post-natal growth and metabolism. We have investigated the metabolic consequences of altered growth hormone signalling in mutant mice that have truncations at position 569 and 391 of the intracellular domain of the growth hormone receptor, and thus exhibit either low (around 30% maximum) or no growth hormone-dependent STAT5 signalling respectively. These mutations result in altered liver metabolism, obesity and insulin resistance.

**Methodology/Principal Findings:**

The analysis of metabolic changes was performed using microarray analysis of liver tissue and NMR metabonomics of urine and liver tissue. Data were analyzed using multivariate statistics and Gene Ontology tools. The metabolic profiles characteristic for each of the two mutant groups and wild-type mice were identified with NMR metabonomics. We found decreased urinary levels of taurine, citrate and 2-oxoglutarate, and increased levels of trimethylamine, creatine and creatinine when compared to wild-type mice. These results indicate significant changes in lipid and choline metabolism, and were coupled with increased fat deposition, leading to obesity. The microarray analysis identified changes in expression of metabolic enzymes correlating with alterations in metabolite concentration both in urine and liver. Similarity of mutant 569 to the wild-type was seen in young mice, but the pattern of metabolites shifted to that of the 391 mutant as the 569 mice became obese after six months age.

**Conclusions/Significance:**

The metabonomic observations were consistent with the parallel analysis of gene expression and pathway mapping using microarray data, identifying metabolites and gene transcripts involved in hepatic metabolism, especially for taurine, choline and creatinine metabolism. The systems biology approach applied in this study provides a coherent picture of metabolic changes resulting from impaired STAT5 signalling by the growth hormone receptor, and supports a potentially important role for taurine in enhancing β-oxidation.

## Introduction

Growth hormone (GH) is both the major regulator of postnatal growth and an important metabolic regulator, influencing many aspects of lipid, carbohydrate, and protein metabolism [1]. GH exerts its anabolic actions by increasing lean body mass and decreasing adiposity. These actions are mediated largely by increased protein synthesis, decreased proteolysis, inhibition of insulin-stimulated adipogenesis and induction of lipolysis [2]–[7]. Treatment with GH also affects hepatic glucose metabolism, mostly through the stimulation of gluconeogenesis [8]. A large number of other physiological processes are affected by GH, including drug and xenobiotic metabolism through the regulation of P450 cytochrome expression [9].

**Figure 1:**
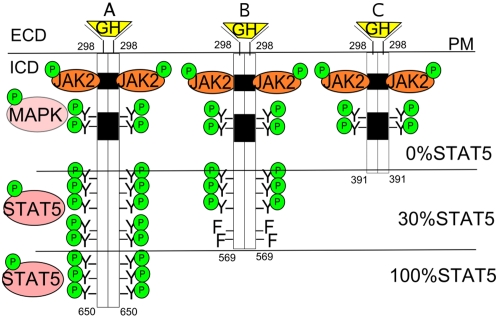
Structure of the intracellular domain of the Growth Hormone Receptor (GHR). The mutations in the GHR have been made in the intracellular domain (ICD) of the receptor. (A) The wild-type has intact signaling through JAK2, MAPK and STAT5. (B) In mutant 569 the ICD has been truncated at residue 569 and two distal tyrosines were mutated to phenylalanines resulting in only 30% of wild-type STAT5 signaling in response to GH. (C) Mutant 391 has been truncated at residue 391 and has no STAT5 signaling ability, while normal JAK2 and MAPK signaling is maintained.

GH acts through its receptor on the cell surface, which is a cytokine class I receptor with multiple tyrosines on the intracellular domain. Binding of the hormone to the receptor induces receptor tyrosine phosphorylation with intracellular signaling through a number of pathways, such as signal transducer and activator of transcription 5 (STAT5), Mitogen-activated protein kinase (MAPK), Phosphoinositide-3 kinase (PI3K) and Janus kinase 2 (JAK2) [10], [11], leading to differential gene expression and changes in physiological response. While the role of GH in metabolism has been studied in a number of animal models and in humans undergoing GH therapy [12]–[16], the contribution of individual pathways to metabolism remains unclear. Treatment of GH-deficient adults or the elderly has been shown to normalize the altered body composition seen in GH deficiency, including increased fat mass, decreased muscle mass and decreased bone mineral density. Transcript changes associated with these metabolic alterations have been studied in animal models, but the full extent and physiological consequences of the altered transcript profiles are not clear [15], [17], [18]. Recently, we have described growth hormone receptor (GHR) mutant mice, with truncations of the intracellular domain of the GHR at position 569 and 391 [19]. These truncations lead to altered signaling through the GHR in response to hormone binding and allow us to study the contribution of particular GH receptor signaling domains to gene expression and metabolism. In particular, these mouse strains exhibit variable levels of STAT5 signalling in response to GH stimulation (Figure 1) and show substantial alterations in hepatic gene expression, together with growth deficit and later onset obesity.

The microarray analysis in our previous study focused on the genes involved in GH enhancement of postnatal growth. We identified sets of genes regulated by particular signaling pathways with a major focus on STAT5 targets. However, there was little analysis of how this differential gene expression affects metabolism in the GHR mutant mice. As GH regulates metabolism in many ways, we were interested in identification of the actual metabolic changes that relate to the development of obesity and insulin resistance in our mice. However, changes in gene expression do not directly measure metabolic changes, and mapping of the differentially expressed genes onto metabolic pathways only provides an indication of pathways that can be affected, without defining the actual metabolic consequences. Therefore there was a need to use an alternative method to assess the global metabolic changes and their time course to determine the likely causes of observed phenotypic changes in our mouse model.

Any significant perturbation of metabolism, such as the one caused by the GHR mutations, is reflected in the composition of body fluids such as urine, blood or saliva, which yield a different “metabolic fingerprint” for each metabolic state [20]. Consequently, valuable information about metabolic changes can be obtained by monitoring global changes in the composition of biofluids of various populations or individuals [20]. The experimental approach enabling this is metabonomics and has been defined as “the quantitative measurement of the dynamic multiparametric metabolic response of living systems to pathophysiological stimuli or genetic modification” [21]. As it is able to analyze global metabolic changes in biofluids it is an appropriate tool for comparing GHR mutant mouse models and allows us to integrate metabolite data with our previous microarray study [19].

Nuclear magnetic resonance (NMR) spectroscopy is one of the major techniques used in metabonomic studies. The advantage of using NMR over other techniques such as mass spectroscopy is that it requires only minimal sample preparation, is non-destructive and inherently quantitative [20]. NMR spectra possess a wealth of metabolic information, containing signals from thousands of individual metabolites that are observed simultaneously and that can partially overlap. Because of the information density of NMR spectra, data are routinely analysed by a combination of data reduction and pattern recognition methods using multivariate statistical analysis, such as principal components analysis (PCA) or partial least squares discriminant analysis (PLS-DA) [22]. This strategy results in identification of groups with similar metabolic patterns. In addition, individual metabolites discriminating between these experimental groups can be identified.

The aim of this study was therefore to identify metabolic changes in our GHR mutant mice by analysis of urinary and hepatic metabolites and to correlate these changes with transcriptome-based pathway analysis to establish metabolic pathways affected by the extents of differential STAT5 signalling. This is to our knowledge the first study linking the analysis of transcriptional changes based on microarrays with metabonomic analysis of actual metabolite changes in strains of receptor mutant mice. Our approach has allowed us to identify key metabolites and altered pathways, which provide a coherent and complementary picture of physiological changes at the level of the whole organism in relation to the metabolic role of GH.

## Results

### Growth hormone receptor (GHR) modifications *in vivo*:

In this study we used animals previously generated in our laboratory with characterized mutations in the intracellular domain of the GHR [19] (Figure 1). Our previous study showed that their hepatic response to GH administration is impaired in relation to the extent of STAT5 signalling, with mutant 569 showing 30% of the wild-type response, while mutant 391 has no STAT5 signalling response [19]. At the same time both mutants have shown normal activation of JAK2, MAPK and STAT3 signalling, and microarray comparison with mice harboring complete GH receptor deletion showed most GH-regulated transcripts do not involve STAT5 signalling. Thus, in this study we determined the metabolic consequences of the loss in GHR signalling from the distal portion of the GH receptor, including total loss of STAT5 signalling.

### Adipose accumulation:

**Figure 2:**
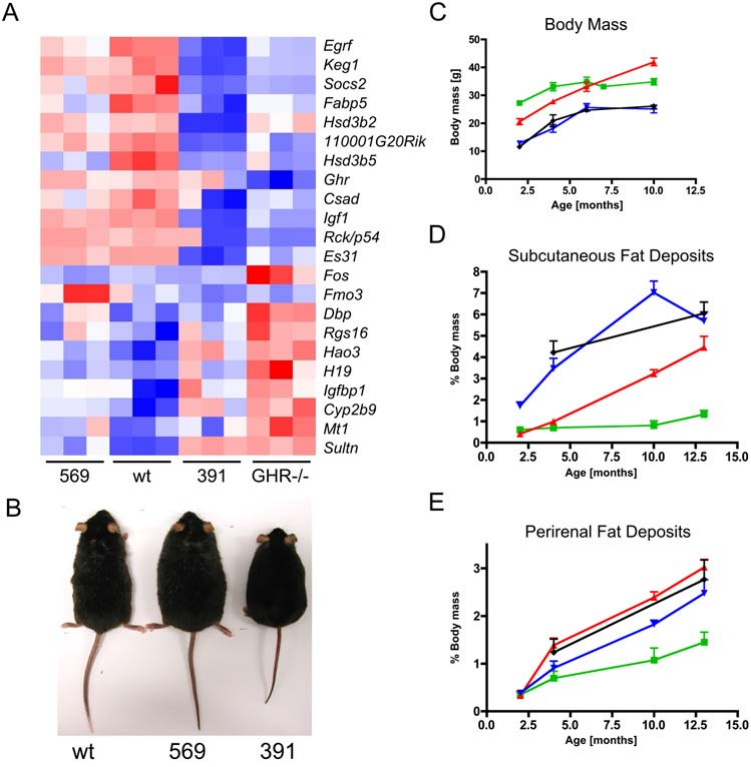
Identification of marker genes differentiating between the groups and physiological characterisation of GHR mutant mice. (A) A heatmap of classifier gene expression in liver tissue of wild-type and GHR mutant mice at 42 days age from the GeneRaVE analysis, clustered according to similarity of expression. Gene expression has been represented as a scale between red and blue, with red indicating over expression and blue representing under expression. Gene abbreviations are used according to current nomenclature. (B) A picture of 10 month old male mice used in this study. (C) The weight curves of male mice from 2 months to 10 months. (D) Subcutaneous fat accumulation of male mice from 2 months to 13 months. (E) Perirenal fat accumulation of male mice from 2 months to 13 months. (C-E) green = wild-type, red = mutant 569, blue = mutant 391, black = GHR−/−.

The most striking physiological and phenotypic change in the mutant animals is a dramatic increase in weight, resulting from obesity (Figure 2B–E). The weight of all mutants and wild-type littermates up to 21 days of age was not significantly different [19]. The body weight differentiated in the following 2 months, and was in both sexes significantly lower for each of the 569, 391 and GHR^−/−^ mutants than for their wild-type littermate controls at 2 months of age ([19], Figure 2C). However, over the next 4 months the weight of the mutant 569 mice increased, so that at 6 months the difference with the wild-type was no longer statistically significant, and later mutant 569 mice became significantly heavier. While the weight of the 391 mutant mice was essentially lower than that of the wild-type at the same time (Figure 2C), they were also significantly shorter than wild-type mice as evident in Figure 2B, and thus became severely obese at a lower body weight. The observed increase in weight reflected an increased fat deposition for all mutant mouse strains. Indeed, when the fat accumulation was analyzed over time at 2, 4, 10 and 13 months (Figure 2D and E), there was a significant difference between the mutants and the wild-type. This difference is especially pronounced in the subcutaneous fat deposits, which is expected, as subcutaneous fat pads have been shown to be the deposit most sensitive to GH regulation in rodents [23]. While the wild-type mice showed only a small increase in subcutaneous fat up to 13 months of age, the 391 mutants began to accumulate subcutaneous fat from early in their life reaching a maximum at 10 months of age (Figure 2D). The 569 mutant showed a slower, more constant increase of subcutaneous fat and by 13 months of age almost reached that of the 391 mutant. The picture changes only slightly when the perirenal fat deposits are viewed (Figure 2E). While the wild-type exhibited only a slow increase in the percentage of dissectable body fat, all mutants accumulated fat very rapidly after 2 months of age, with the 569 mutant displaying a rate of perirenal fat accumulation that was similar to the GHR−/− mutant and slightly faster than the 391 mutant (Figure 2E).

### Metabolic pathway analysis and identification of marker genes in microarray data

To investigate the biological processes underlying this dramatic change in phenotype, we determined the effects of the GHR mutations and altered STAT5 signalling on transcript expression in liver tissue of 42-day old mice, In a first round of analysis, we used the MAS 5.0 algorithm and fold changes with a cut-off of 1.5-fold to identify differentially expressed genes. These were subsequently analysed by ANOVA to identify predictor genes able to separate the four classes of microarray data (wild-type, mutant 569, 391 and GHR^−/−^) [19]. This generated a list of 20 genes regulated concordantly in all mutant mice and additional 31 genes differentially expressed between the individual mutant groups. However, as this analysis was a supervised approach, we wanted to use an unsupervised multivariate statistical method similar to the tools used in metabonomic analysis. In a second round of analysis we therefore used GeneRaVE as a method to identify the classifier genes differentiating the groups and explore gene interactions using network analysis.

GeneRaVE identified three genes as differentiating the groups, *RCK/p54*, *Hsd3b5 and Es31*, whose expression levels can separate the four classes with 84% accuracy. Subsequent classifier genes from 11 rounds of selection were extracted from the dataset and are shown in Figure 2A. Among these were transcripts increased in the wild-type mice, such as cysteine sulfinic acid decarboxylase (*Csad*), esterase 31 (*Es31*), hydroxysteroid dehydrogenase-5, delta<5>-3-beta (*Hsd3b5*), hydroxysteroid (17-beta) dehydrogenase 2 (*Hsd3b2*) and kidney expressed gene 1 (*Keg1*) as well as transcripts decreased in the wild-type, such as sulfotransferase family 2A, dehydroepiandrosterone (DHEA)-preferring, member 2 (*Sult2a2*), flavin containing monooxygenase 3 (*Fmo3*), hydroxyacid oxidase (glyoxylate oxidase) 3 (*Hao3*), cytochrome P450, family 2, and subfamily b, polypeptide 9 (*Cyp2b9*). The only genes not identified as biomarkers in our previous approach were *Ghr*, *Rck*, *Fmo3* and *Dbp*. In addition, *Hsd3b2*, *Fos*, *H19*, *Mt1* and *Igfbp1*, were differential in our previous study [19], but not reported.

Based on these results we predicted taurine as one of the metabolites that would differentiate between the groups, as the mRNA levels of the key enzyme involved in its biosynthesis, *Csad*, were reduced in mutant 391 and GHR^−/−^. In contrast, the possible metabolic effects of changes in genes encoding other enzymes were not immediately obvious. It was also clear from this analysis that at 42 days of age the hepatic expression pattern of the mutant 569 was very similar to that of the wild-type, and it was expected that these mice would have very similar metabolic profiles.

GeneRaVE [24] identified several sets of genes that could be used to discriminate the different strains of mice under study. Individually they represent classifiers, but taken together they represent a group of genes whose expression has been altered in the different mouse strains in comparison to the wild-type. However, this still did not clarify which biological pathways and processes are affected in the GHR mutant mice.

To further characterize the pathways affected in the mutant mice and to determine how the physiological response was changed, we used Gene Ontology analysis and pathway mapping. Gene Ontology (GO) analysis using NetAffx GO Browser identified that 228 (57.3%) out of 398 genes differentially expressed between the strains were involved in metabolism, with a number of biological processes identified. The most prominent processes affected were generation of precursor metabolites and energy metabolism, lipid metabolism, nucleic acid metabolism, biopolymer metabolism and sulfur metabolism (Table 1). In addition to these metabolic processes and those identified in [19], inflammatory response was identified as significantly altered. Further breakdown of affected biological processes was possible using the DAVID Functional Annotation Tool [25], which identified 55 biological processes with a p-value<0.05 and a minimum of 4 genes present. The 15 top biological processes altered in GHR mutant mice as identified by DAVID are shown in Table 2.

**Table 1:** Gene Ontology analysis of differentially expressed genes (equal or >1.5-fold) using NetAffx Gene Ontology Browser.

Biological Process	% of genes involved	Number of genes	Total number of genes in process	Probability value*
Generation of precursor metabolites and energy	9.6	54	562	2e–16
Lipid metabolism	8.6	50	577	1.7e–12
Biopolymer metabolism	1.8	65	3531	5.1e–11
Catabolism	7.5	41	542	7.6e–8
Nucleic acid metabolism	1.9	51	2599	1.2e–6
Inflammatory response	11	12	109	1.6e–5
Carbohydrate metabolism	5.6	22	391	0.02

* Probability value – the likelihood of this over-representation by chance.

**Table 2:** Top 15 metabolic processes identified among the differentially expressed metabolic genes using DAVID Functional Annotation Tool.

	Biological Process	Number of genes	%	p-value
1	generation of precursor metabolites and energy	47	10.8	1.95e–11
2	electron transport	34	7.8	1.4e–9
3	steroid biosynthesis	12	2.8	3.3e–6
4	catabolism	35	8	1.8e–5
5	cellular lipid metabolism	29	6.6	2.2e–5
6	biosynthesis	57	13.1	2.5e–5
7	coenzyme metabolism	17	3.9	2.7e–5
8	lipid metabolism	32	7.3	2.7e–5
9	lipid biosynthesis	18	4.1	4.1e–5
10	steroid metabolism	15	3.4	4.7e–5
11	cellular catabolism	29	6.6	1.2e–4
12	cofactor metabolism	17	3.9	1.3e–4
13	sulfur metabolism	9	2.1	2.1e–4
14	general metabolism	209	47.9	4.2e–4
15	cellular biosynthesis	48	11	4.4e–4

While gene ontology (GO) analysis and functional annotation were pointing consistently to differences in metabolic responses between the mouse groups, they did not indicate which individual pathways were affected. Therefore we used pathway mapping to identify these. In addition to functional classification, DAVID mapped the genes successfully to pathways (Table 3). In addition, we also performed pathway mapping using Pathway Miner (http://www.biorag.org). This analysis indicated major changes in multiple pathways, including xenobiotic metabolism, complement cascades, glutathione, tricarboxylic acid (TCA) cycle, fatty acid metabolism and others (Table 3). It is clear that some pathways were identified with both tools, while others were only identified with one. One of the reasons for such discrepancies is that both tools have not managed to map many of the differentially expressed genes. DAVID has mapped only 33% of input genes to the pathways in the Kyoto Encyclopedia of Genes and Genomes (KEGG) data base, while Pathway Miner mapped 17% of input genes to the KEGG pathways. This highlights the insufficiencies in analysis using pathway mapping and GO analysis and a need for an alternative method of analysis of metabolism.

**Table 3:** Pathways in the Kyoto Encyclopedia of Genes and Genomes (KEGG) database analysed using DAVID Functional Annotation Tool and Pathway Miner.

Pathway	DAVID			Pathway Miner		
	Number of genes	%	p-value	569	391	GHR^−/−^
Metabolism of xenobiotics	12	2.8	8.8e–5	4.4e–3	9.9e–4	4.3e–3
Complement and coagulation cascades	13	3	8.9e–5	3.5e–4	7e–5	1e–4
Gluthathione	9	2.1	3.04e–4	2.6e–3	4.3e–3	1.3e–3
TCA	7	1.6	9.2e–4	ns	1.7e–2	1.3e–2
Fatty Acid metabolism	8	1.8	2.8e–3	Not identified		
Valine, Leucine and isoleucine	8	1.8	2.8e–3	ns	3e–2	ns
Androgen and estrogen metabolism	7	1.6	3.7e–3	Not identified		
Proteasome	6	1.4	1.5e–2	Not identified		
Arachidonic acid metabolism	8	1.8	1.9e–2	ns	4.5e–3	ns
Oxidative phosphorylation	11	2.5	2e–2	ns	1.8e–2	0
Propanoate metabolism	5	1.1	3.4e–2	ns	1.6e–2	ns
Glycine, serine and threonine metabolism	6	1.4	3.9e–2	ns	3.5e–2	1.6e–2
Lysine degration	6	1.4	4.7e–2	Not identified		
Bile acid biosynthesis	5	1.1	4.9e–2	2.1e–2	4.5e–3	ns

ns: non significant

NMR-based metabonomics provides this alternative, as it integrates the responses of all different organs to the altered GHR signalling on a whole-body level and thus offers a direct, cost-effective and non-invasive avenue to monitor actual metabolic changes in a subject over several months. Therefore we used NMR metabonomics to provide a global metabolite analysis of the GHR mutant mice.

### NMR spectra of mouse urine

**Figure 3:**
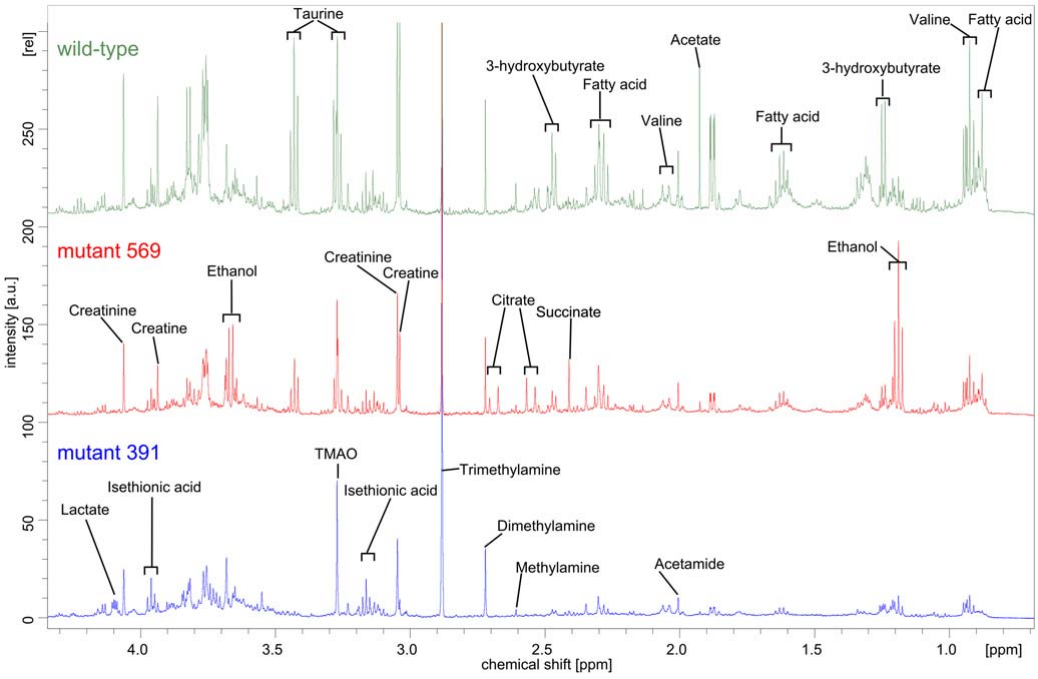
1D proton NMR spectra of mouse urine at 298 K. Spectra of one individual of four months age from each mouse strain are shown. Top: wild-type, middle: 569 mutant, bottom: 391 mutant. The identity of relevant metabolites is indicated above each spectrum.

500 MHz one-dimensional (1D) proton NMR spectra of urine from wild-type mice (n = 16) and GHR mutant mice (n = 39) were compared. Urine from GHR^−/−^ mice was not analyzed with NMR spectroscopy because of difficulties in obtaining samples from those animals. Only a few systematic differences were detected by visual inspection, as can be seen in the comparison of urine spectra at four months of age, depicted in Figure 3. The chemical compounds present in the urine samples were assigned on the basis of previously published chemical shift data [26], [27], data available in small metabolite data bases (http://mdl.imv.liu.se, http://www.hmdb.ca, http://www.bmrb.wisc.edu), and additional information obtained from high-resolution homo- and heteronuclear two-dimensional (2D) correlation spectra of representative urine samples of the wild-type and 391 mouse strains. To facilitate chemometric analysis and to reduce the complexity of the NMR spectra, all 1D spectra were data reduced to integral segments (“buckets”) with a width of 0.05 ppm.

### Principal Components Analysis of urinary data

**Figure 4:**
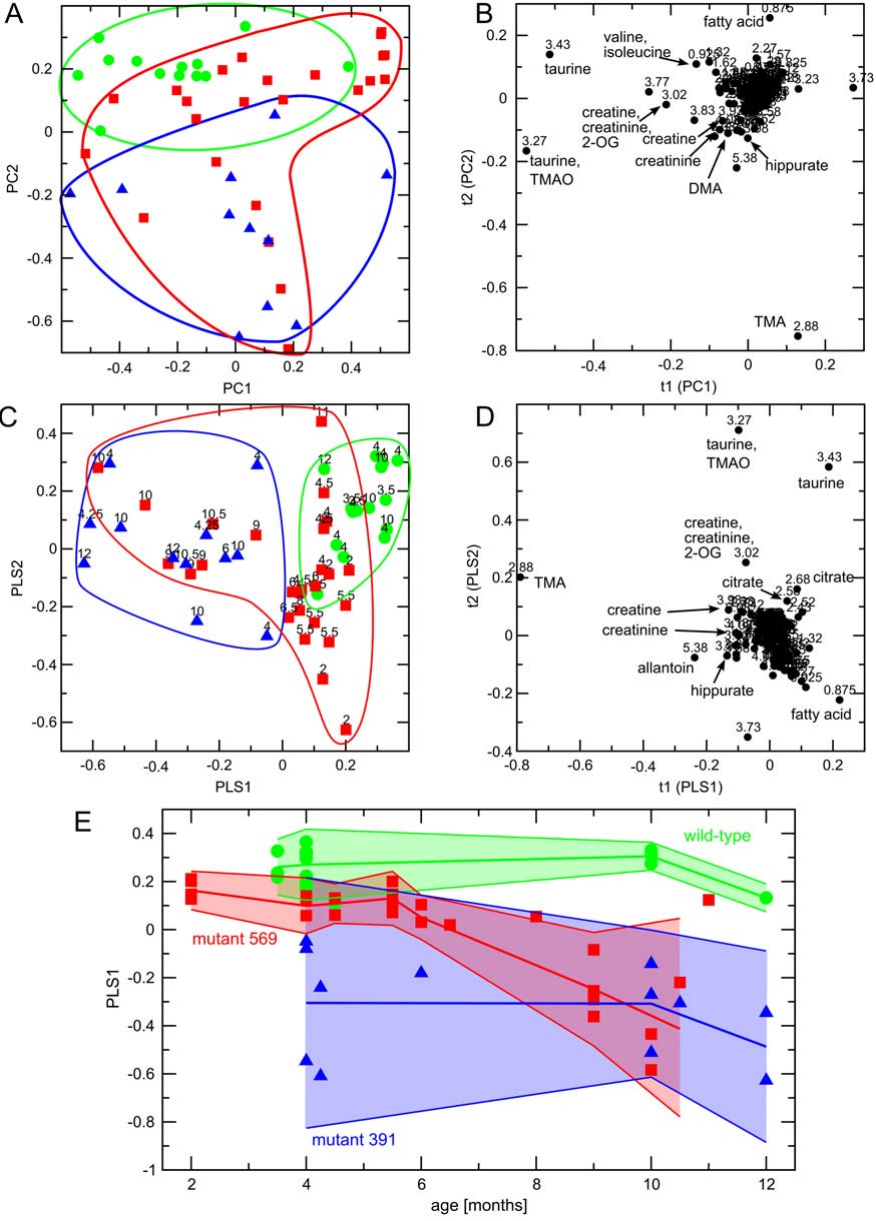
Statistical analysis of metabolites in mouse urine. (A) *Principal components analysis - scores plot of PC1 versus PC2.* Each data point represents one mouse urine sample, and the distance between points in the score plot is an indication of the similarity between samples. Green circles: wild-type, red squares: 569 mutants, blue triangles: 391 mutants. The borders of the groups are also highlighted by lines in the corresponding colors. (B) *Principal components analysis - loadings plot of PC1 versus PC2.* Each data point represents one bucket (with the chemical shift indicated explicitly). The plot indentifies which spectral regions (and thus which chemical compounds) are responsible for the differences between the spectra observed in the scores plot. The loadings coefficients in each dimension are correlation coefficients that indicate how strongly each metabolite is correlated with the observed variance in the respective dimension. t = 0 means no correlation, and t = 1 means total correlation. Several significant metabolites are indicated explicitly. 2-OG: 2-oxoglutarate, DMA: dimethylamine, TMA: trimethylamine, TMAO: trimethylamine-N-oxide. The model consists of 7 PCs and represents data from 48 samples. Regions containing water, urea, and ethanol signals were excluded from the PCA. (C) *Partial Least Squares-Discriminant Analysis - scores plots of PLS1 versus PLS2.* The coding of groups is same as in panel (A). (D) *Partial Least Squares-Discriminant Analysis - loadings plot of PLS1 versus PLS2.* The chemical shift of each bucket as well as selected metabolites are indicated explicitly. Abbreviations as in panel (B). (E) *Metabolic trajectories of the three mouse strains.* Depicted is the change in the first PLS component PLS1 with the age of the mice. The mean metabolic trajectories (obtained by averaging the PLS1 scores of mice with similar age) are indicated by thick lines. The wild-type trajectory is colored green, the 569 mutant red, and the 391 mutant blue. The 2σ standard deviation from each mean trajectory is indicated by areas shaded in the respective color. As can be seen, the metabolic trajectory of the 569 mutant mice moves with age from the position of the wild-type mice to the position of the 391 mutant mice.

Principal components analysis (PCA) is a standard technique of pattern recognition and multivariate data analysis. The technique is particularly suited to the analysis of a set of data where each individual measurement contains itself a multitude of data – as is the case in a series of NMR spectra, containing signals from hundreds of individual metabolites. PCA compares all measurements with each other and explains the variation inherent in the data set in terms of artificial components called principal components (PCs). The first principal component (PC1) is reponsible for the largest amount of variation in the data, the second principal component is orthogonal to PC1 and explains the largest amount of yet unexplained variation, and so forth with higher PCs. In effect, the first two PCs explain the vast majority of variation present in the data. In the resulting scores plot (Figure 4A), each data point corresponds to one NMR spectrum, and the position in the plot is determined by the difference of that spectrum with respect to the average of the respective PC. Essentially, the closer points are to each other the more similar the corresponding spectra are. This feature allows one to recognise easily if the data form several distinct groups or are all similar to each other. In the corresponding loadings plot (Figure 4B) each data point corresponds to one spectral region ( = bucket), and the position in the plot is equivalent to the correlation coefficient of that bucket with the corresponding PC. This means that it is possible to identify from the loadings plot which spectral regions and thus which chemical compounds are responsible for any grouping of data points observed in the scores plot. Loadings coefficients ( = correlation coefficients) of 0 mean no correlation, and loadings coefficits of 1 mean total correlation is observed between a particular bucket ( = chemical compound) and the variation in the corrresponding dimension. As can be seen from Figure 4B, the loadings coefficients for most spectral regions cluster around zero, indicating that concentration changes in the metabolites corresponding to these regions are insignificant. However, a number of spectral regions are clear outliers in the loadings plot, indicating that concentration changes in the corresponding metabolites are significantly associated with the variation/separation observed in the scores plot in the corresponding PC. Due to their nature as correlation coefficients the loadings coefficients are not directly associated with fold changes. They rather indicate, which concentration changes are significant, irrespective of the actual magnitude of change.

PCA was performed on urine to find if it was possible to distinguish the three different mouse strains on the basis of their NMR spectra. A preliminary PCA showed that the urine samples were contaminated by ethanol, possibly from sterilisation of the urine collection vials (data not shown). Thus, a second data reduction and PCA was performed, in which the spectral regions containing ethanol signals were excluded. The PCA scores plot of the first two principal components (Figure 4A) showed considerable clustering of the three groups, albeit with some overlap between the groups. Notably mice from the 391 strain were clearly separated from wild-type mice, with mice from the 569 strain exhibiting a distribution in between these two extremes. The separation between the three groups was mostly along PC2.

### Partial Least Squares-Discriminant Analysis of urinary data

To maximise separation between the three mouse strains a partial least squares-discriminant analysis (PLS-DA) was performed. In contrast to PCA, which is an unsupervised multivariate statistical classification method that works to explain maximum variation between samples, PLS-DA is a supervised method that explains maximum separation between pre-defined classes (*e.g.* genotype or health state) in the data. Thus, the class membership of each sample must be known and is provided in form of a Y-table. PLS-DA then performs a regression of the original data against the Y-table to maximise separation between the individual classes. In our case, the group identity of the three mouse strains was used in the Y-table. The PLS-DA scores plot of the first two partial least squares (PLS) components, PLS1 and PLS2, (Figure 4C) showed improved separation of the three mouse strains compared to the PCA. Again, mice from the 391 strain are completely separated from the wild-type strain, with mice from the 569 strain exhibiting a distribution in between these two extremes. The separation between the three groups was mostly along PLS1, suggesting that this PLS component contains metabolites associated with differences between the three strains.

The equivalent loadings plot showed several spectral regions that exhibit significant correlation with PLS1 and/or PLS2 (Figure 4D). The chemical compounds corresponding to the NMR signals in these regions were identified as described above.

The components most strongly associated with PLS1 and PLS2 are taurine, trimethylamine (TMA) and trimethylamine-N-oxide (TMAO). Taurine was less prominent in the spectra of the 391 and 569 mutants than in the wild-type mice. In contrast TMA and TMAO were present in higher concentrations in the spectra of the mutant mice as compared to the wild-type. Other compounds discriminating between the three strains were creatine, creatinine, allantoin, and hippurate, all of which increased in the mutant mice. In contrast, the concentrations of citrate and 2-oxoglutarate were decreased in the mutant mice. Other compounds with significant loadings coefficients in PLS1 or PLS2 that could be identified are listed in Table 4 and indicated in Figure 4B and D.

**Table 4:** Identified metabolites with significant loadings coefficients in the first three PLS components t1 to t3.

Metabolite	^1^H chemical shifts [ppm] multiplicity / ^13^C chemical shifts[ppm]^a^	Increase (+) or decrease (−) in:			
		m./wt^b^ ^c^	569/wt^c^	391/wt^c^	391/569^c^
Taurine	3.43t / 35.6; 3.27t / 47.5	−−	−−	−−	−−
Trimethylamine (TMA)	2.88s / 44.6	++	++	++	−−
Hippurate	7.84d / 122; 7.64t / 127; 7.56t / 123; 3.97d / 43.8	++	+	++	++
Allantoin	7.36m / 124; 5.39s / 63.6	++	0	++	++
Creatinine	4.06s / 56.2; 3.05s / 30.1	+	+	++	++
Creatine	3.94s / 53.9; 3.04s / 36.8	+	+	+	+
Isethionic acid	3.96t / 57; 3.17t / 52.7	+	+	+	+
TMAO	3.28s / 59.5	−−	0	−−	−−
Citrate	2.69d / 44.5; 2.55d / 44.5	−	−	−	−
2-Oxoglutarate (2-OG)	3.02t / 29.9	−	−−	−	−
Oxaloacetate	3.37s / 48.7	−−	0	0	0
Succinate	2.41s / 34.1	−	−	0	0
Dimethylamine (DMA)	2.72s / 34.4	0	+	0	−
Methylamine	2.61s / 24.7	0	0	0	0
3-Hydroxybutyrate	2.45t / 44.2; 1.25d / 21.9	−	−	−	0
Fatty Acid	2.30t / 35.6; 1.64m, 1.63m, 1.61m / 24.9; 1.60m, 1.59m; 0.88t / 15.2	−−	−	−−	++
Valine	2.06m / 26.6; 0.94d / 21.5	−	−	−	0
Isoleucine	0.93t / 12.8	−	−	−	0

a chemical shifts in ppm denote the position of a signal in the NMR spectra, the multiplicity describes the coupling pattern of a NMR signal, s: singlet, d: doublet, t: triplet, m: multiplet. These data were used for metabolite identification.

b m.: mutants, wt: wild-type.

c t1 to t3 are the loadings coefficients ( = correlation coefficients) for each metabolite in the first three PLS-DA dimensions. t = 0 means no correlation between metabolite and variance in the respective dimension, and t = 1 means total correlation. For the majority of metabolites, t is between −0.1 and 0.1. ++: t>0.2, +: 0.1<t<0.2, 0: −0.1<t<0.1, −: −0.2<t<−0.1, −−: t<−0.2.

In addition to the PLS-DA comprising all three mouse genotypes (Figure 4C–D), two-way PLS-DAs were performed with all possible pairwise comparisons of the genotypes (data not shown). These analyses confirmed that especially the mutant 391 strain is completely separated from the wild-type mice, while the 569 mutant mice are more similar to the 391 mutant mice than the wild type especially with age. As expected, essentially the same metabolites that are responsible for the differences between all three genotypes were with minor differences also significant for discriminating between groups in the two-way comparisons (as shown in Table 4).

Interestingly, while the positions of the wild-type mice and the 391 mutants in the PLS-DA scores plot remain constant, the position of the 569 mutant mice is clearly dependent on the age of the mice: The samples of the younger 569 mutant mice with an age up to 6.5 months cluster in the PLS1-PLS2 scores plot in the metabolic space close to the wild-type mice (Figure 4C), while the urine samples of older 569 mice between 9 and 12 months of age cluster in the area of the 391 mutant mice. From the average PLS scores of mice of similar age it was possible to construct metabolic trajectories for the three mouse strains as shown in Figure 4E. This graph reveals the same picture with even grater clarity: The metabolism of the wild-type and 391 mutant mice remains essentially unchanged with age, while the metabolism of the 569 mutant mice changes from a wild-type like metabolism in young mice to a 391 mutant-like metabolism after 6 months of age, associated with the onset of obesity.

### Principal Components Analysis of murine liver tissue

After establishing the effects of the GHR mutations on the metabolite profile of mouse urine we wanted to determine if these mutations manifest in the metabolite profile of liver, as this was the subject of the microarray analysis and a major GH target tissue. We specifically wanted to compare the taurine status in the livers of wild-type mice to their counterparts from the 569 and 391 mutants, as taurine status relates to obesity [28] (see below) . In addition, we included obese wild-type animals fed on a high-fat diet to distinguish effects caused by obesity from the effects caused by the GHR mutations.

**Figure 5:**
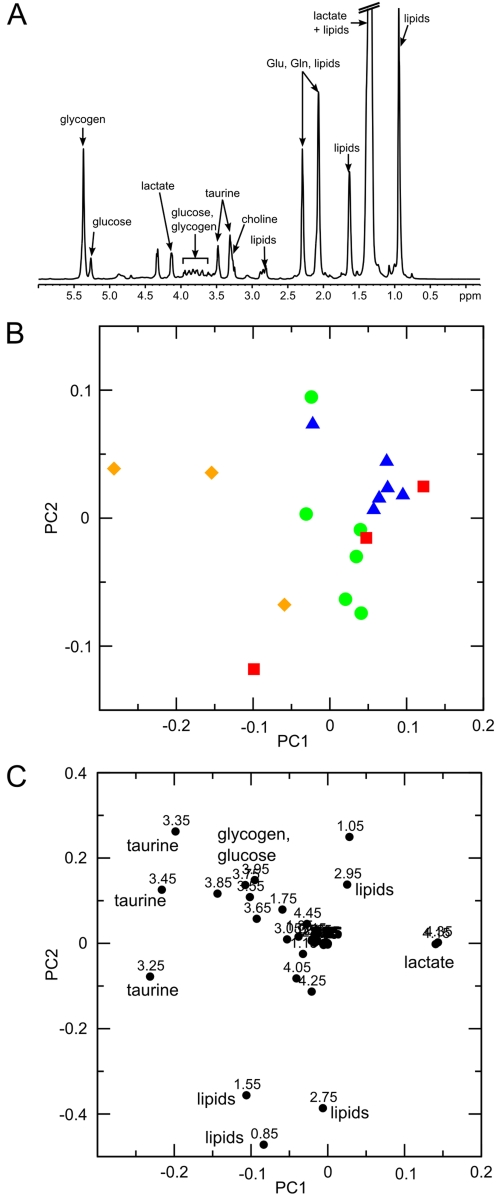
Statistical analysis of metabolites in murine liver tissue. (A) *1D proton HR-MAS NMR spectrum of murine liver tissue.* The identity of relevant metabolites is indicated. Glu: glutamate, Gln: glutamine (B) *Principal components analysis - scores plot of PC1 versus PC2.* Each data point represents one liver tissue sample. Green circles: wild-type, red squares: 569 mutants, blue triangles: 391 mutants, orange diamonds: wild-type on high-fat diet. (C) *Principal components analysis - loadings plot of PC1 versus PC2.* Each data point represents one bucket (with the chemical shift indicated explicitly). Several significant metabolites are indicated explicitly. The model consists of 3 PCs and represents data from 18 samples. Regions containing water, lactate and glutamate/glutamine signals were excluded from the PCA.

700 MHz 1D high-resolution magic angle spinning (HR-MAS) proton NMR spectra of intact liver tissue from four months old wild-type mice (n = 6), mutant 569 mice (n = 3), mutant 391 mice (n = 6), and wild-type mice fed on a high-fat diet (n = 3) were measured. An example spectrum is shown in Figure 5A.

A preliminary PCA of liver tissue spectra was dominated by the signals of lactate and glutamate/glutamine, which changed disproportionately in intensity and chemical shift (data not shown). Thus, a second data reduction and PCA was performed, in which the spectral regions containing these signals were excluded. The PCA scores plot of PC1 *versus* PC2 (Figure 5B) showed considerable clustering of the four groups of mice, albeit with some overlap between them. As is the case in the spectra of mouse urine, the 391 mutant mice were clearly separated from wild-type mice, with the 569 mutants exhibiting a distribution encompassing these two extremes. The spectra of the high-fat diet wild-type mice also form a separate group, distinct from both the lean wild-type and 391 mutant mice. The separation between these groups was mostly along PC1. The loadings plot (Figure 5C) reveals that the buckets associated with taurine (δ = 3.20–3.50 ppm) are the most significant descriptors associated with this group separation. It indicates that on average taurine levels are decreased in the liver tissue of the 391 mutant mice as compared to the wild-type. This parallels the result obtained in the multivariate analysis of mouse urine. In contrast, taurine levels are increased in obese wild-type mice fed on a high-fat diet when compared to their lean counterparts (Figure 5BC).

## Discussion

In this paper we have shown that metabonomics can identify significant metabolic changes in animal models exhibiting altered signalling by the growth hormone receptor and that these changes correlate with altered expression of key metabolic enzymes in the liver, the major GH target organ. While both metabonomics and microarray approaches are very different, both allowed identification of groups of animals according to their genotype. This was achieved by using multivariate statistical tools, which identified markers (metabolites and genes) correlating with a particular genotype. As the strains of mice used in this study have the same C57Bl/6J background and differ only in the GHR sequence [19] (Figure 1), the metabolic differences observed can be exclusively attributed to the effects induced by mutations in the GHR associated with differential signalling in response to GH. While the altered transcripts identified by microarrays are useful, it was important to understand how they affect the physiological outcome of the mutations in the GHR mutant mice. This aim was achieved by combining Gene Ontology analysis, pathway mapping and metabonomic data to generate a global image of metabolic changes in these mice (Supplementary Information Figure S1).

The observed separation of the mouse strains in PCA, PLS-DA and GeneRaVE, reflects the observed phenotype and gene expression, with the 569 mutant locating between the wild-type and the 391 mutant. The 569 mutant, possessing only partial STAT5 signalling ability, was generally not well separated from the wild-type or the 391 mutant. Moreover, while the metabolism in mutant 391 is clearly different from that of the wild-type littermates (Figure 4E) at 4 months of age and stays different up to 12 months of age, the metabolism of the 569 mutant mice changes with the age of the mice. The younger 569 mutant mice with an age up to 6 months have a metabolic fingerprint close to the wild-type mice (Figure 4E), which is consistent with their microarray profiling at 42 days of age (Figure 2A). On the other hand, the metabolic fingerprint in the older 569 mice between 9 and 12 months has changed to resemble the 391 mutant mice (Figure 4E). At this time they also became visibly obese (Figure 2B). It should be noted that metabolic enzymes are often regulated by means other than transcription, for example substrate availability, enzyme half-life, changes in activity, or binding co-factors [29]. Thus, an analysis of predicted metabolic changes based on microarray data alone does not provide the full picture, especially when changes in physiology are subtle or appear later in life. Characterisation of metabolite levels are a more direct measure of the actual metabolic changes, and it is recognised that metabonomics provides a connection between differential mRNA responses and the metabolic phenotype [30]–[33].

The NMR-metabonomic analysis identified taurine as the most prominent metabolite able to distinguish between wild-type and mutant mice, with urinary taurine levels being decreased in mutants. This was in agreement with the microarray analysis, which identified *Csad* (cysteine sulfinic acid dehydrogenase) as one of the marker transcripts decreased in the mutant mice. As CSAD is the rate-limiting enzyme in taurine biosynthesis [34], the decreased transcript levels are concordant with the reduced taurine levels observed in the metabonomic analysis (Supplementary Information Figure S1). The observed change is also consistent with reduction in the STAT5 signalling in GHR mutant mice, as *Csad* transcript levels were decreased in STAT5A^−/−^ mice [35]. Taurine is the best example of the convergence of the two methods and the power of multivariate statistics in this combined analytical approach.

Taurine is an important metabolite involved in the bile acid synthesis pathway [34], osmoregulation and intracellular calcium levels [36]. It is generally regarded as a sensitive marker indicating changes in the liver metabolism [37]. Our microarray analysis has identified altered metabolic transcripts in the liver, in particular in lipid, energy, nucleic acid and sulphur metabolism. Moreover, our previous and current analysis of the 569 mutant mice showed that they develop obesity and insulin resistance later in life [19]. The change observed in the metabolic profile of mutant 569 is accompanied by an increase in fat depots (Figure 2D–E). Taurine is related to the development of obesity, as its conjugation to bile acids is the only mechanism of excretion of cholesterol, and thus reduces serum levels of cholesterol in humans, in particular in low-density lipoproteins (LDL) [38]. Decreased taurine levels will thus lead to increased cholesterol retention. This links taurine biosynthesis with the cholesterol and steroid metabolism, which are also affected in the GHR mutant mice (Kerr, Lichanska, Rowland, Waters, in preparation). In addition, taurine levels are decreased in diabetic patients [39].

Most significantly, a vicious cycle involving obesity and taurine has recently been discovered [28], implicating low levels of taurine in the continuing maintenance of this condition. The concentration of taurine in the blood regulates positively the expression of cysteine deoxygenase (CDO), one of the key enzymes involved in taurine biosynthesis (Supplementary Information Figure S1, schematic), in white adipose tissue. Thus, decreasing blood taurine levels lead to less CDO, further decreasing the taurine concentration. Importantly, taurine promotes fatty acid oxidation (β-oxidation) and increases mitochondrial energy metabolism. A depletion of taurine blood levels, resulting from deficient STAT5 generation would thus reduce β-oxidation, promoting further obesity and creating the vicious cycle [28] (Supplementary Information Figure S1, schematic).

High expression levels of *Csad* are found only in the liver and kidney [40], [41], while they are negligible in most other organs, especially in muscle and adipose tissue, making any contributions from transcript changes in these organs to the observed reduction in urinary taurine levels highly unlikely. It is likely that both liver and kidney contribute to urinary taurine under the control of GH, since both tissues possess responsive GH receptors. Indeed, the metabonomic analysis of liver tissue shows the same changes in taurine levels as seen in the urine, *i.e.* taurine levels are reduced in liver tissue of 391 mutant mice when compared to lean wild-type mice (Figure 5BC). This result demonstrates directly that the reduced urinary taurine levels in the mutant mice reflect decreased hepatic taurine biosynthesis. In contrast, taurine levels are increased in obese (fat fed) wild-type mice compared to their lean counterparts (Figure 5BC). This shows that the effects of GHR mutation are clearly different from the effects of obesity and that the obesity in the mutant mice is likely to be a consequence of the low taurine levels and the GHR mutations rather than the converse. It is plausible that the increase in hepatic taurine levels seen in fat-fed wild-type mice is a compensatory mechanism to overcome the increased supply of dietary triglycerides by promoting β-oxidation.

Indeed, while a reduction in β-oxidation would be expected in obesity, we have observed an increase in expression of a number of genes involved in fatty acid β-oxidation. Similar observations were made previously by other groups [15], [17], [18] in animals deficient in GH. Most of these transcripts are normalized by GH treatment. The changes observed by ourselves and others are generally small, 1.5–2 fold between the wild-type and mutants or GH-deficient animals, and may reflect the hepatic response to increased lipid load resulting from upregulation of the CD36 transporter and lipoprotein lipase expression evident in the microarray analysis (19), leading to steatosis in older animals.

The increased lipid transport in the GHR mutant mice is likely to create an increased demand for lecithin, which can be satisfied by increased lecithin synthesis via glycine and choline, causing an increased flux through the choline metabolism. This interpretation is corroborated by increased urinary levels of TMA, dimethylamine (DMA) and TMAO. Indeed, these three metabolites are the second-most significant metabolites in the metabonomic analysis after taurine. They can be made either via endogenous pathways from choline or from dimethylglycine (Supplementary Information Figure S1), or via exogenous pathways involving gut flora [42], [43]. The increased flux through the endogenous glycine-choline pathway is further substantiated by increased transcriptional levels of FMO3 [44], [45], the enzyme catalysing the conversion of TMA to TMAO, and this enzyme was identified as one of our marker genes (Figure 2A).

The increased flux through the choline metabolism is likely to be further facilitated by the decreased taurine production. Due to the feedback inhibition of CDO and the decreased levels of *Csad* levels, the taurine precursor cysteine can be shunted through alternative pathways, by being converted via serine to glycine and then further into choline and lecithin as outlined above.

However, the metabonomic data suggest that the majority of the cysteine is shunted into the biosynthesis of creatine, which undergoes breakdown to creatinine (Supplementary Information Figure S1). The urinary levels of both compounds were increased, which can indicate an overflow of the serine to glycine pathway, alteration of muscle metabolism, kidney function, or all of those. Indeed it has been previously suggested that increased cysteine synthesis is associated with hypercreatinuria [46]. Creatine is also an important metabolite of energy metabolism because a phosphorylated form of it (phosphocreatine) is able to phosphorylate ADP to ATP [47], thereby generating energy reserves. The excretion of creatinine is a sensitive marker for lean muscle mass [48], which is in turn highly influenced by GH, and thus it could be argued that the observed changes in creatine/creatinine excretion are a result of changes in the muscle mass as result of the disrupted GH signaling. However, the percentage of lean muscle mass decreases in the GHR mutant mice, while excretion of creatine and creatinine increases, meaning that the first interpretation is more likely.

We anticipated abnormal energy metabolism, as 21% of differentially expressed metabolic genes are involved in generation of energy and precursor metabolites. The NMR analysis demonstrated changes in the urinary levels of TCA cycle metabolites, with decreased urinary concentrations of citrate, succinate, oxaloacetate and 2-oxoglutarate. These changes can have a variety of reasons and are not straightforward to interpret, as TCA cycle metabolites are also central metabolic branching points for biosynthesis or breakdown of fatty acids as well as for biosynthesis and breakdown of amino acids. A reduction in urinary citrate can also indicate metabolic acidosis [49]. Correlating with the metabonomic observations, the microarray analysis indicated alterations in the TCA cycle, albeit at low levels. GH is known to regulate a number of TCA cycle enzymes, in particular NADH-dependent isocitrate dehydrogenase, succinate CoA ligase and fumarate hydratase [15], [18].

One of the other categories changed in Gene Ontology analysis was the metabolism of nucleic acids and its components (Table 1). While we were unable to match the changes in gene expression to a single pathway, there were indications of changes in pyrimidine and to smaller extent in purine metabolism. The metabonomic data showed increased urinary levels of allantoin, which is an intermediate in purine metabolism. This is most likely the result of an increased flux in purine metabolism, possibly due to increased DNA breakdown and excretion via the xanthine-allantoin-urea pathway. Alterations in this pathway would not be surprising, as it has been described previously that in GH-deficient patients there is increased excretion of urea [50], [51] in fasting individuals, which is overcome by GH replacement. Relevant to this is a large increase in transcript levels of hydroxylamine oxidase (*Hao3*), for which transcript levels were increased from 3.8 fold in mutant 569 to 11 fold in mutant 391 and GHR^−/−^ mice. This would lead to an increase in nitrite levels and consequently ammonia levels, which can enter the urea cycle, further increasing urea excretion.

### Conclusions

In this paper we have shown that truncations in the intracellular domain of the GHR that impair STAT5 signaling in particular cause dramatic metabolic changes, leading to obesity, and involving the metabolism of the whole body, as evidenced by their consistency between liver tissue and urine. The most prominent hallmarks of these changes are a decrease in taurine levels and changes in choline metabolism, characterized by increased levels of TMA, TMAO and DMA. Both changes are connected via lipid transport pathways and amino acid metabolism and support a potentially important role of taurine in regulating β-oxidation.

We have also has shown how various parts of metabolism interact with each other in response to altered hormonal levels. The effects of GH on metabolism were always described as complex, but they can be unraveled when global metabolism analysis methods are used.

The multivariate statistical techniques analysing metabonomic and gene expression data implicated the same biochemical pathways. The convergence of both methods on the same biochemical pathways underlines the complementarity of both techniques and exemplifies the power of this combined approach. Our results demonstrate that metabonomic data can be successfully linked with microarray results to develop a coherent picture of physiological changes in response to a varying genetic background, allowing a deeper understanding of systems biology. Future studies combining more detailed genetic expression profile data with established data of metabolic fluxes in tissues, metabolic modelling, and metabonomic data will be useful in developing a more detailed understanding of how changes in gene transcription lead to the observed metabolic and systemic changes.

## Materials and Methods

### Animals

Animals were housed in an approved facility and treated as per university guidelines with ethics approval from the University of Queensland Animal Ethics Committee and the Australian Office of the Gene Technology Regulator. Water and standard feed pellets were available *ab libitum* under a 12 hr light/dark cycle at 20–22°C. Prior to urine collection, animals were fasted overnight (16 hours), with water being provided *ab libitum.* Urine samples were taken from 55 male mice aged from 2 to 12 months. The mice were either wild-type C57Bl/6J (n = 16), or had a truncation in the GHR at lysine 569 together with conversion of tyrosines 539 and 545 to phenylalanine (mutant 569) (n = 27) or had a truncation in the GHR at lysine 391 (mutant 391) (n = 12). In addition, three animals/strain were used for gene expression analysis using microarrays (see below). In addition, a second cohort of male mice was used for metabonomic analysis of liver tissue. This cohort comprised six wild-type C57Bl/6J mice fed on standard chow, three wild-type mice fed on a special high-fat diet, three mutant 569 mice and six mutant 391 mice, both fed on standard chow. Mice were sactrificed at four months of age, the left lateral lobe of the liver was removed immediately after killing, snap frozen in liquid nitrogen, and stored at −80°C until required. Creation of the two mutant strains is described in Rowland *et al.* [19] and of the GHR^−/−^ mice in Zhou *et al.* [52].

### Adipose tissue analysis

Adipose tissue was dissected from two separate areas. Subcutaneous fat pads were obtained from the side of the back legs and body, and renal adipose tissue was dissected from around the kidneys. Each of the fat pads was weighed, and their weight relative to the whole body weight is reported in the figures. For each time point 4–8 animals/strain were sacrificed.

### Microarray analysis

Mice were sacrificed at 42 days of age and the livers were dissected directly into RNAlater solution (Ambion, Austin TX). Total RNA was extracted using RNAqueous kit (Ambion) according to the manufacturer's instructions, quality of RNA was confirmed by spectrophotometry and gel electrophoresis. Probes were prepared as described in Rowland *et al.* [19]. Three animals were used in each group. The samples were hybridized to the Affymetrix U74v2A arrays.

The increases and decreases, as well as signal log ratios (SLRs, equivalent of fold changes) were identified following standard Affymetrix protocol with MAS 5.0, and then the comparisons were loaded into DMT (Affymetrix), which allowed identification of transcripts changing in the same direction and the number of comparisons in which they change as described in [19]. In this analysis a gene was scored as significantly changed in one group in comparison to the other if it was changed in the same direction in at least 8 out of 9 comparisons performed and the fold change was above 1.5-fold. We used Anova to test the statistical significance of the array data with a cut-off score of p<0.0005 and obtained a set of 398 genes differentially expressed between the three groups.

### Marker identification using microarrays

Microarray data were normalised using RMA [53] with qspline normalisation of perfect match probes only. Median polish of the results was used for a final expression value per probeset. The resulting expression values were then analysed with GeneRaVE algorithms [24], made available via the RChip library for the R statistical language package, (RChip library and vignette are available from https://www.bioinformatics.csiro.au/GeneRave/index.shtml). Genes were identified whose expression values could be used to distinguish between the sets of array data. These genes were removed from the dataset and a further set of classifiers were obtained. This iterative process was continued until the predictive power of the dataset was exhausted (11 rounds). Default settings for GeneRaVE were used (kbess & bbess), as described in the accompanying software vignette. The function ‘deleteRepeat’ was used to extract multiple classifiers from the data using the HGmultc method for fitting a multiclass logistic regression models to our data. Ten-fold cross validation was also used to assess the error associated with the chosen classifiers.

Functional classification and pathway analysis was performed on the 399 genes identified in the previous study [19] as differentially expressed. Gene Ontology analysis using NetAffx GO Browser, and DAVID Functional Classification Tool [54] were used to identify the main functional groups over-represented among the differentially expressed genes. Pathway mapping was performed using DAVID and the Pathway Miner (http://www.biorag.org) [55]. For DAVID and NetAffx GO Browser we have uploaded a gene list, in form of Affymetrix GeneIDs, of 399 differentially expressed genes, Biorag actually required GeneBank accession numbers and log ratio changes for the 3 groups.

Array data are available from the NIH Gene Expression Omnibus database (http://www.ncbi.nlm.nih.gov/geo/), with accession numbers GSM15488, GSM15489, GSM15490, GSM15491, GSM15492, GSM15493, GSM15494, GSM15495, GSM15496, GSM15497, GSM15498, and GSM15499.

### Preparation of urine samples

Urine was collected from animals into glass containers and frozen immediately at −20°C. Samples were prepared for NMR by diluting 200 μl mouse urine with 300 μl 0.1 M sodium phosphate buffer, pH 7.4, and 50 μl D_2_O. In cases, where less than 200 μl mouse urine was available, urine was diluted with H_2_O to 200 μl. Samples contained trimethylsilylpropionic acid (TSP) for calibration of the ^1^H and ^13^C chemical shifts.

### NMR spectroscopy of urine samples

NMR spectroscopy of mouse urine samples was carried out on a Bruker AV500 NMR spectrometer equipped with a 5 mm self-shielded z-gradient triple resonance probe and a sample changer. All spectra were recorded at 298 K. 1D proton spectra were measured with 256 scans and 64k resolution over a spectral width of 14 ppm. To ensure solvent suppression of the water signal, 1D spectra were measured with the noesypr1d pulse program (Bruker pulse program library), using a mixing time of 150 ms. The water signal was additionally suppressed by low-power continuous irradiation on the water resonance during the mixing time and the relaxation delay of 2.3 s.

In addition, the following 2D spectra were recorded on representative samples of wild-type and 391 mice to facilitate the assignment of signals in the 1D spectra: total correlation spectroscopy (TOCSY), double-quantum filtered correlation spectroscopy (DQF-COSY), ^13^C-heteronuclear single-quantum correlation (^13^C-HSQC), ^13^C-heteronuclear multiple bond correlation (^13^C-HMBC) and ^13^C-HSQC-TOCSY.

In all 2D spectra the ^1^H carrier frequency was positioned on the water resonance. TOCSY [56] and DQF-COSY [57] spectra were recorded for each sample with a resolution of 4096×512 points and a spectral width of 14.0 ppm. Quadrature detection in the indirect dimension was achieved using the time-proportional phase incrementation (TPPI) [58] method. Water suppression in the DQF-COSY experiments was achieved using selective low-power irradiation of the water resonance during the relaxation delay of 1.0 s. For the TOCSY experiments a 3-9-19 WATERGATE scheme [59] was used employing gradient pulses of ∼6 Gcm^−1^ either side of a 10 kHz 3-9-19 binomial pulse. TOCSY experiments used a MLEV17 sequence [60] of 80 ms duration for isotropic mixing.

^13^C-HSQC [61], [62] spectra were recorded with a resolution of 4096×256 data points. The spectral width was 14 ppm for ^1^H and 100 ppm for ^13^C with the ^13^C carrier frequency positioned at 45 ppm. ^13^C-HSQC-TOCSY [63] spectra were measured with 4096×256 data points, 128 scans, and spectral widths of 14 ppm and 200 ppm in ^1^H and ^13^C, respectively. Isotropic mixing was achieved with a DIPSI2 sequence [64] of 80 ms duration. The ^13^C-HMBC [65], [66] spectra were measured with 4096×256 data points, 128 scans, and spectral widths of 14 ppm and 200 ppm in ^1^H and ^13^C, respectively. A relaxation delay of 2.0 s was used, and the transfer delay was optimized for indirect coupling constants of 6 Hz.

### HR-MAS NMR spectroscopy of liver tissue

For each sample about 50 mg of liver tissue were placed in a 4 mm reduced volume 50 μl zirconia rotor with a teflon top insert and a Kel-F rotor cap (Bruker Biospin, Germany). All experiments were carried out on a Bruker AV700 spectrometer, equipped with a 4 mm HR-MAS triple resonance probe with z-gradients, at a sample spinning rate of 8 kHz. 1D spectra were aquired with the standard cpmgpr1d pulse program, using a rotor-synchronised Carr-Purcell-Meiboom-Gill sequence [90°-(τ-180°-τ)_n_-acquisition] of 20 ms duration (n = 80) to suppress signals from macromolecules and other substances with short T_2_ values. The refocusing delay τ was set to 125 μs to match the rotation speed of the rotor. Proton 1D spectra were measured unlocked with 128 scans and 32k resolution over a spectral width of 15 ppm. The water signal was additionally suppressed by continuous low-power irradiation on the water resonance during the relaxation delay of 3.0 s. The total acquisition time for each spectrum was 11 min.

### Processing of spectra

All NMR spectra were processed with TopSpin (Bruker Biospin GmbH, Rheinstetten, Germany). 1D spectra were processed to a size of 64k after multiplying with an exponential window function. Spectra were manually phase and baseline corrected, and chemical shifts were referenced to the TSP signal in the case of urine spectra or lactate in the case of HR-MAS spectra of liver tissue.

Homonuclear 2D spectra were processed to a size of 4096×1024 data points. Prior to Fourier transformation, a Lorentz to Gauss transformation with a line broadening factor of of −10 Hz and a GB of 0.1 was applied in the direct dimension, while in the indirect dimension data were linearly predicted, using 30 poles, and then multiplied with a squared sine bell function, shifted by π/2.

Heteronuclear 2D spectra were processed in a similar fashion to a data matrix size of 2048×512 data points, except that the window function in the direct dimension was a squared sine bell function, shifted by π/2. A magnitude calculation was applied in the indirect dimension to all ^13^C-HMBC spectra after Fourier transformation

### Data reduction

The 1D spectra of mouse urine were data reduced over the shift range of δ = 10.0–0.5 ppm into spectral integral regions (“buckets”) of 0.05 ppm width, using AMIX3.6.6 (Bruker Biospin GmbH, Rheinstetten, Germany). The regions δ = 4.6–5.0 ppm and δ = 5.5–6.5 ppm were excluded to avoid artefacts from varying water suppression and cross-saturation through chemical exchange in the urea signal. NMR signals were integrated for each region and normalised to the total spectral intensity over the whole spectrum. A second bucket table was constructed in a similar fashion, additionally excluding the regions of 1.22–1.16 ppm and 3.7–3.6 ppm to exclude ethanol signals.

A similar procedure was applied for the HR-MAS spectra of mouse liver tissue. Buckets ranged from δ = 10.0–0.2 ppm with a bucket width of 0.1 ppm. The region δ = 4.5–6.5 ppm was excluded to avoid artefacts from varying water suppression. A second bucket table was constructed in a similar fashion, additionally excluding the regions of δ = 1.9–2.5 ppm, and δ = 1.2–1.5 ppm to avoid artefacts from shifts in the signals of lactate and glutamate/glutamine that otherwise dominated the analysis.

### Principal components analysis (PCA)

PCA was performed in AMIX, using the data from the bucketed 1D spectra of mouse urine. A first PCA model was constructed using all samples. The scores plots of PC1 versus PC2 and PC1 versus PC3 were inspected for differences between the different mouse strains. Samples that constituted outliers were identified in AMIX by cross-validation, removed from the model, and a further round of PCA was performed. The refined model contained seven principal components (PCs) and comprised data from 44 urine samples.

A second PCA model was constructed in a similar fashion from the bucket table, additionally excluding ethanol signals. The final model contained seven PCs, explaining 90.8% of total variance, and comprised data from 48 urine samples.

PCA was also performed similarly with the data from the bucketed 1D spectra of mouse liver tissue. The PCA model contained all samples and three PCs. As this analysis was dominated by large variations in the signals of lactate and glutamate/glutamine, a refined PCA was performed, additionally excluding these signals. The final model contained three PCs, explaining 92.0% of total variance, and comprised data from 18 tissue samples.

### Partial Least Squares Discriminant Analysis (PLS-DA)

PLS-DA was performed in AMIX, using data from the bucketed 1D spectra of urine and group IDs of 0 (wild-type mice), 1 (569 mice), and 2 (391 mice) as Y table. Buckets containing signals of water, urea and ethanol were excluded from the analysis as described above. Samples that constituted outliers were identified in AMIX by cross-validation, removed from the model, and further round of PLS-DA was performed. The refined model comprised data from 54 urine samples and contained seven PLS components, explaining 84.2% of total X-variance and 87.5% of total Y-variance with an rmsec value of 0.249. In addition, the mouse strains were compared pairwise in three separate PLS-DAs. These analyses were performed similarly to the PLS-DA comparing all three mouse strains.

Metabolic trajectories for each mouse strain were constructed by averaging the PLS1 scores for groups of mice with similar age. Age stages where only one single mouse was available were included in the next closest age group if their ages did not differ by more than half a month, otherwise they were omitted from the analysis. In addition, the 2σ standard deviation from the mean PLS1 scores was calculated to indicate the possible bandwidth of the metabolic trajectories.

### Identification of metabolites

Metabolites were identified in the NMR spectra by comparing their ^1^H and ^13^C chemical shifts and coupling patterns with corresponding values of metabolites from previously published data [26], [27] and from publicly accessible data banks, such as the BioMagRes data bank (http://www.bmrb.wisc.edu), the Metabolomics Database of Linkoping (MDL) (http://mdl.imv.liu.se), or the Human Metabolome Data Bank (http://www.hmdb.ca). In addition, the information contained in the high-resolution 2D NMR spectra (proton-proton couplings, direct and indirect proton-carbon couplings) was used for the identification of metabolites.

## Supporting Information

Figure S1 *Metabolic Pathways affected by GHR mutations.* Individual enzymes are depicted in italics, and sections of metabolism in bold and boxed. Metabolites with increased concentration in the mutants, as detected by the metabonomic study, are indicated in green and marked by plus signs, whereas metabolites with decreased concentration in the mutants are indicated in red and marked by minus signs. Enzymes in the pathways with increased or decreased expression levels are indicated similarly. The feedback loops between taurine levels, CDO levels, β-oxidation and obesity are indicated by dashed connections in blue. Arrowheads indicate positive feedback and bar heads negative (inhibitory) feedback. The two negative feedback connections beween taurine and fat levels form a vicious cycle. Enzyme abbreviations: *Csad* - cysteine sulfinic acid decarboxylase, *Pdha1* - pyruvate dehydrogenase alpha 1, *Idh2* - isocitrate dehydrogenase 2, *Suclg1* - succinate-CoA ligase, *Sdhb* - succinate dehydrogenase complex, subunit B, *Fmo3* - flavin containing monooxygenase 3, LCAT - lecithin cholesterol acyltransferase, and CDO - cysteine dioxygenase 1.
(0.63 MB TIF)
