# Supplementary figures and images for: Altered Metabolism of Growth Hormone Receptor Mutant Mice: A Combined NMR Metabonomics and Microarray Study
Source: PLoS One. 2008 Jul 23;3(7):e2764. doi: 10.1371/journal.pone.0002764 (PMC2447874; doi:10.1371/journal.pone.0002764)

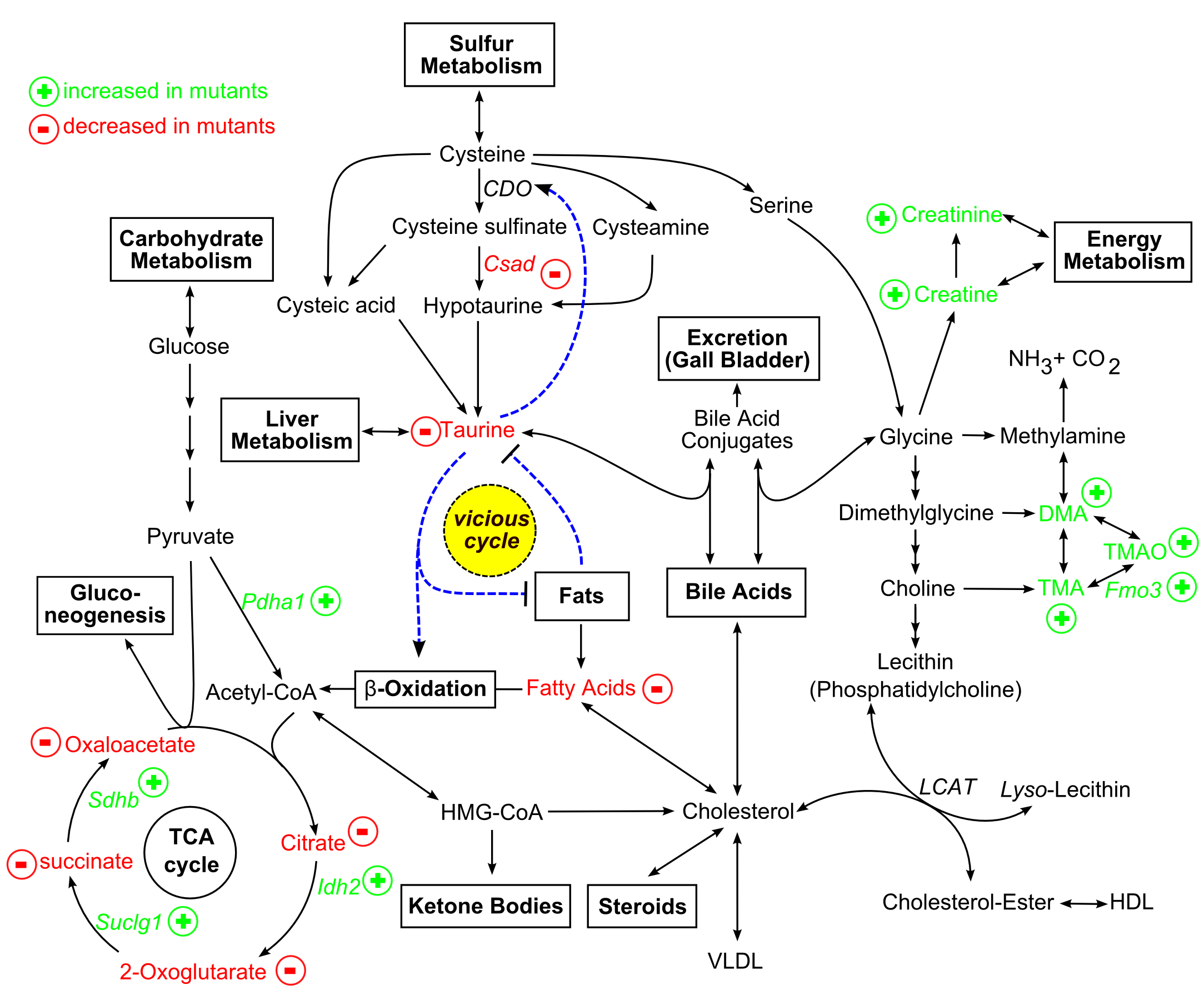

Supplement: Figure S1 — Metabolic Pathways affected by GHR mutations. Individual enzymes are depicted in italics, and sections of metabolism in bold and boxed. Metabolites with increased concentration in the mutants, as detected by the metabonomic study, are indicated in green and marked by plus signs, whereas metabolites with decreased concentration in the mutants are indicated in red and marked by minus signs. Enzymes in the pathways with increased or decreased expression levels are indicated similarly. The feedback loops between taurine levels, CDO levels, β-oxidation and obesity are indicated by dashed connections in blue. Arrowheads indicate positive feedback and bar heads negative (inhibitory) feedback. The two negative feedback connections beween taurine and fat levels form a vicious cycle. Enzyme abbreviations: Csad - cysteine sulfinic acid decarboxylase, Pdha1 - pyruvate dehydrogenase alpha 1, Idh2 - isocitrate dehydrogenase 2, Suclg1 - succinate-CoA ligase, Sdhb - succinate dehydrogenase complex, subunit B, Fmo3 - flavin containing monooxygenase 3, LCAT - lecithin cholesterol acyltransferase, and CDO - cysteine dioxygenase 1. (0.63 MB TIF) [file pone.0002764.s001.tif]
